# Partner Effects on Depressed Mood in Caregiving Dyads Are Most Pronounced in Cancer Caregiving, Partner Caregiving

**DOI:** 10.1093/geroni/igaa057.1132

**Published:** 2020-12-16

**Authors:** Nadia Al Nassar, Kristin Litzelman

**Affiliations:** University of Wisconsin-Madison, Madison, Wisconsin, United States

## Abstract

Theory and empirical evidence indicate that the well-being of caregivers and their care recipients is interrelated, although conflicting evidence has emerged across different caregiving populations. To establish a more nuanced understanding of this phenomenon, we used data from the National Health and Aging Trends Survey and the National Survey on Caregivers (2015 and 2017, n=759 dyads with complete longitudinal data) to construct actor-partner interdependence models assessing how spillover of depression varies by care recipient health condition (cancer, dementia, stroke, diabetes, or other conditions) and relationship type (spouse/partner, child, or other). Across condition types, the largest magnitude partner effects were observed in dyads with cancer, in which a one-point increase in caregiver depressed mood was associated with a 0.23-point increase in subsequent care recipient depressed mood (p=0.02) and a one-point increase in care recipient depressed mood was associated with a 0.33-point increase in subsequent caregiver depressed mood (p<0.01). Moderation by cancer status was statistically significant (pinteraction=0.03). Among spouse/partner caregivers, caregivers’ depressed mood was associated with subsequent depressed mood in the care recipient (p<0.05) but there was no evidence of spillover from the care recipient to the caregiver. Conversely, in both adult child caregivers and other caregivers, there was evidence for spillover from the care recipient to the caregiver (p<0.05) but not the reverse. The findings show that the interrelationship in the well-being of caregivers and care recipients varies by key caregiving characteristics, with implications for the development, dissemination, and implementation of interventions targeting caregiver, care recipient, and dyadic well-being.

